# Personality Traits and Cyberbullying Perpetration Among Chinese University Students: The Moderating Role of Internet Self-Efficacy and Gender

**DOI:** 10.3389/fpsyg.2022.779139

**Published:** 2022-03-22

**Authors:** Wei Xu, Shujie Zheng

**Affiliations:** ^1^School of Educational Science, Ludong University, Yantai, China; ^2^Institute for Education and Treatment of Problematic Youth, Ludong University, Yantai, China; ^3^School of Humanities and Social Sciences, Binzhou Medical University, Yantai, China

**Keywords:** personality traits, cyberbullying, Internet self-efficacy, gender, university students

## Abstract

Cyberbullying is a serious global problem that affects many teenagers and university students. Recent studies have explored the relationship between personality traits and cyberbullying, but the mechanism needs further research. This paper examines the impact of personality traits on cyberbullying perpetration of Chinese university students and the moderating role of Internet self-efficacy (ISE) and gender. By random cluster sampling, 549 university students (45.7% boys) participated in filling out the self-report questionnaires. The results revealed: (1) conscientiousness, agreeableness, and openness were significantly negatively correlated with cyberbullying perpetration. (2) The stratified regression showed that ISE moderated the relation between agreeableness and cyberbullying. Gender moderated the relationship between agreeableness and cyberbullying, and openness and cyberbullying.

## Introduction

The 48th China Statistical Report on Internet Development shows that the internet penetration rate of China has reached 71.6%. By June 2021, the average online time of Chinese netizens was 26.9 h per week, and instant messaging users accounted for 97.3% of all netizens (China Internet Network Information Center [CNNIC], 2021). Unfortunately, this has also led to more cyberbullying, which is also known as online aggression. Cyberbullying is defined as an aggressive and intentional act carried out by an individual or a group of people, through electronic forms of contact to harm another person over a period of time (Smith, 2015). It differs from traditional bullying because it allows anonymity in online platforms such as email, chat rooms, instant messaging apps and websites. Examples of cyberbullying include harassment, masquerading, denigration, flaming, and intimidating (Smith, 2015). Multiple past studies have consistently reported that cyberbullying had significant deleterious effects on the victims such as loneliness, anxiety, depression, low self-esteem, substance abuse and even suicide (Bauman et al., 2013; Gámez-Guadix et al., 2013; Varghese and Pistole, 2017; Kowalski et al., 2019; Martínez-Monteagudo et al., 2020).

Given the negative psychological impacts on individuals, it is important to identify predictors of cyberbullying. The factors have been classified into four main categories, for example, personal, socio-cognitive, psychological, and environmental factors (Shaikh et al., 2020), among which the influence of personality traits (psychological factor) and gender (personal factor) is undeniable.

Previous studies have revealed a close connection between personality traits and cyberbullying, including self-esteem, impulsivity, the dark triad, and the Big Five (Zhou et al., 2019; Lei et al., 2020; Pascual-Sanchez et al., 2021; Schade et al., 2021). The Big Five model can capture the commonality of most existing personality trait systems at a broad level of abstraction, and it would be useful in organizing results of cyberbullying research from an integrated perspective. The Big Five include neuroticism, conscientiousness, agreeableness, openness, and extraversion, of which high scores on neuroticism are generally seen as negative, while high scores on the last four dimensions are generally seen as positive. Neuroticism was found positively correlated with cyberbullying (Çelik et al., 2012), while conscientiousness was found negatively correlated with cyberbullying (Çelik et al., 2012; Alonso and Romero, 2017). The correlation between extraversion and cyberbullying was not significant in most studies. Van Geel (2017) and Zhou (2019) only found a negative correlation between agreeableness and cyberbullying. A new survey on children aged 10–13 showed that only openness was the protective factor of cyberbullying (Escortell et al., 2020).

The inconsistent results may be due to the fact that the tests on the relationship between the Big Five and cyberbullying were carried out in different age groups and different cultural backgrounds. Therefore, a further investigation among Chinese college students is a continuing concern within the field. In addition, the mechanism of the relationship between personality traits and cyberbullying is also worthy of further study. General Aggression Model (GAM) stated that both individual and situational factors play a role in cyberbullying. In studying individual behaviors, Caprara et al. (2010) suggested that personality would also interact with a person’s beliefs in his or her ability to achieve certain behaviors. Anderson and Bushman (2018) added that personality of an individual can influence online aggression through the individual’s internal state such as cognition, affect and arousal. Among various social cognitive factors, ISE may be a moderating variable worth considering.

Internet self-efficacy (ISE) is defined as the user’s belief in his ability to use the Internet (Tsai and Tsai, 2003; Tsai et al., 2011). Unlike face-to-face bullying, cyberbullying, using electronic media to harm others, relies on online knowledge, skills, and networking technology. Existing studies have also found that cyberbullying is related to computer skills, online time, the use of social networks sites, or communication technologies (Monks et al., 2016; Li et al., 2018; Barlett et al., 2019; Kim and Faith, 2019). Studies have shown that ISE was positively correlated with cyberbullying (Xiao and Wong, 2013; Musharraf et al., 2018, 2019). This means that the higher the individual’s ISE, the stronger the motivation to use the network to deal with tasks. Individuals with stronger self-efficacy believe that they can bully others on the Internet, so they may have more bullying behaviors in the network environment. Bussey et al. (2015) found that self-efficacy moderated the relationship between moral disengagement and cyberbullying. The study suggested that self-efficacy alone might not predict cyberbullying, but it did play a role when moral disengagement was involved. Individuals with different personality traits bullying others on the Internet will be affected by ISE. Neurotic individuals have the characteristics of depression, anxiety, hostility, and anger, and high neuroticism is a positive predictor of cyberbullying (Çelik et al., 2012), while high ISE may enhance the positive relationship. On the contrary, agreeableness is a positive personality trait with characteristics such as kindness, cooperation and tolerance. The protective effect of high agreeableness on cyberbullying may be weakened due to high ISE. Thus, it is plausible that individuals with different personality traits could also be moderated by ISE when engaging in cyberbullying.

Gender is also an important predictor of cyberbullying. Currently, there is no consensus on how gender can have influence on cyberbullying. Some studies claimed that boys have more cyberbullying behaviors (Huang and Chou, 2010; Wong et al., 2018; Zhou et al., 2019; Adebayo et al., 2020), while others revealed that there was actually no gender difference in cyberbullying (Kowalski and Limber, 2013). Gender may be another important moderating variable worth considering. Wang et al. (2016) found that gender played a moderating role between moral disengagement and cyberbullying in adolescents, and the relation was stronger in boys than in girls. Similarly, Kircaburun et al. (2018) recently reported that Machiavellianism was strongly associated with cyberbullying perpetrated by male university students, but not females. Therefore, we proposed that the relationship between personality traits and cyberbullying perpetration is stronger in boys than in girls.

### The Present Study

Based on past studies, there is evidence to suggest that both personality traits, ISE and gender play a role in cyberbullying. Understanding the nature of the relationship between these variables will prove helpful in exploring the intervention to curb this issue. Unfortunately, past studies on the relationship between these variables have been ambiguous and there is clearly a lack of understanding of the mechanism involved, In addition, due to cross-cultural differences (Li, 2008; Jin et al., 2019; Park et al., 2021), it is necessary to examine cyberbullying among Chinese university students. Thus, this study intends to explore the moderating role of gender and ISE in the relationship between personality traits (neuroticism, conscientiousness, agreeableness, openness and extraversion) and cyberbullying among undergraduate students in China. The hypotheses in this study are:

To summarize the above discussion, we proposed the following hypothesis:

H1: Neuroticism is significantly positively correlated with cyberbullying (H1a), agreeableness and conscientiousness are negatively correlated with cyberbullying (H1b).

H2: ISE moderated the relationship between neuroticism and cyberbullying, the positive relationship between neuroticism and cyberbullying was enhanced with the increase of ISE (H2a). ISE moderated the relationship between agreeableness and cyberbullying, the negative relationship between agreeableness and cyberbullying was weakened with the increase of ISE (H2b).

H3: Gender moderated the relationship between personality traits and cyberbullying, the relation tend to be stronger in boys than in girls.

## Materials and Methods

### Participants

Using random cluster sampling, 600 freshmen to seniors were selected from a university in Shandong province as the research objects. 51 invalid questionnaires were excluded, and 549 valid questionnaires were obtained. The effective recovery rate was 91.5%. Among the participants, 251 (45.7%) were male, 298 (54.3%) were female, 148 (27.0%) were freshmen, 135 (24.6%) were sophomores, 114 (20.8%) were juniors, and 152 (27.7%) were seniors.

### Measures

#### Big Five Personality Traits

The Chinese Big Five Personality Inventory Brief Version (CBF-PI-B) was used to assess personality traits. The questionnaire, developed by Wang et al. (2010a,b, 2011), contains 40 questions in five dimensions of neuroticism, conscientiousness, agreeableness, openness, and extraversion. Each item is based on a six-point scale that ranges from 1 (strongly disagree) to 6 (strongly agree). The reliability and validity of CBF-PI-B have been tested (Fan et al., 2013; Wang et al., 2018; Zhang et al., 2019). In the current study, Cronbach’s α was 0.85. Internal consistency was 0.82 for neuroticism, 0.78 for conscientiousness, 0.76 for agreeableness, 0.82 for openness and 0.81 for extroversion.

#### Internet Self-Efficacy

The ISE Questionnaire for university students (Liu, 2005) comprises 20 items and is rated on a 5-point Likert scale ranging from 1 (total nonconformity) to 5 (total conformity). The reliability and validity have been tested (Huang et al., 2013; Jiang et al., 2016; Ge et al., 2018). The overall score is obtained by summing all the items. A higher score indicates a higher level of ISE. In this study, Cronbach’s α was 0.95.

#### Cyberbullying Perpetration

The self-report scale (Xu, 2016) consists of 12 questions, using a 5-point scale ranging from 1 (never) to 5 (always). Participants answered questions based on their experiences “in the past semester.” The reliability and validity have been tested (Feng et al., 2016; Ye et al., 2016; Zheng et al., 2017). A sum of the scores on all items represents an overall score on that scale and higher scores indicate higher levels of cyberbullying perpetration. The present study Cronbach’s α was 0.92.

#### Procedure

The investigation was approved by the university ethics committee of the first author. Participants were asked to fill out an online questionnaire, followed by the Big Five Personality Scale, the Cyberbullying Scale and the ISE Scale. Anonymity was guaranteed. Participants were voluntary and could withdraw from the study at any time.

#### Data Analysis

SPSS 22.0 was used for data analysis. we performed the descriptive statistics, correlation analysis of variables and the analysis of moderating effects.

## Results

### Correlation Analysis Among Variables

The descriptive statistics and correlation analysis of variables are shown in Table 1. As the results indicate, the association between neuroticism and cyberbullying was not significant (*r* = 0.08, *P* = 0.069); conscientiousness, agreeableness, and openness were negatively correlated with cyberbullying (*r* = −0.27, *r* = −0.36, *r* = −0.20, *P* < 0.01). Neuroticism has a significant negative correlation with ISE (*r* = −0.22, *P* < 0.01), while conscientiousness, agreeableness, openness, and extroversion have a significant positive correlation with ISE (*r* = 0.36, *r* = 0.24, *r* = 0.45, *r* = 0.39, *P* < 0.01). There was no significant correlation between ISE and cyberbullying (*r* = −0.04, *P* > 0.05).

**TABLE 1:** Descriptive statistics and correlations among variables.

	M	SD	1	2	3	4	5	6	7
1. Neuroticism	25.52	6.77	—						
2. Conscientiousness	33.02	5.83	−0.16**	—					
3. Agreeableness	34.46	5.82	−0.17**	0.47**	—				
4. Openness	32.53	6.12	−0.09*	0.53**	0.44**	—			
5. Extroversion	29.65	6.54	−0.31**	0.43**	0.41**	0.52**	—		
6. Internet self-efficacy	69.66	13.84	−0.22**	0.36**	0.24**	0.45**	0.39**	—	
7. Cyberbullying	17.64	6.89	0.08	−0.27**	−0.36**	−0.20**	−0.05	−0.04	—

*P < 0.05, **P < 0.01.

### The Moderating Effect of Internet Self-Efficacy

The independent and moderating variables are centered on the mean, then, the interaction terms derived from the two variables are calculated. A stratified regression was used to test the moderating effect of ISE (Frazier et al., 2004; Wen et al., 2005). The first step was to put in conscientiousness and ISE, and the second step was to put in the interaction term of conscientiousness and ISE. The same procedures were used to test the moderating effect of ISE on agreeableness, openness, and cyberbullying.

The results showed that the interaction between conscientiousness and ISE was not significant, so is the relationship between openness and ISE. while the interaction between agreeableness and ISE was significantly positive (*B* = 0.01, *t* = 2.75, *P* < 0.01), with an independent impact of 1% on cyberbullying, see Table 2.

**TABLE 2:** The moderating effect of Internet self-efficacy (ISE) on agreeableness and cyberbullying.

	Cyberbullying					
	Step 1			Step 2		
	*B*	*t*	*P*	*B*	*t*	*P*
Agreeableness	−0.44	−8.95	<0.001	−0.45	−9.20	<0.001
Internet self-efficacy	0.02	1.08	0.28	0.02	0.86	0.39
Agreeableness ×Internet self-efficacy				0.01	2.75	0.006
Δ*R*^2^	0.13			0.01		
Δ*F*	40.59		<0.001	7.55		0.006
						

**FIGURE 1:**
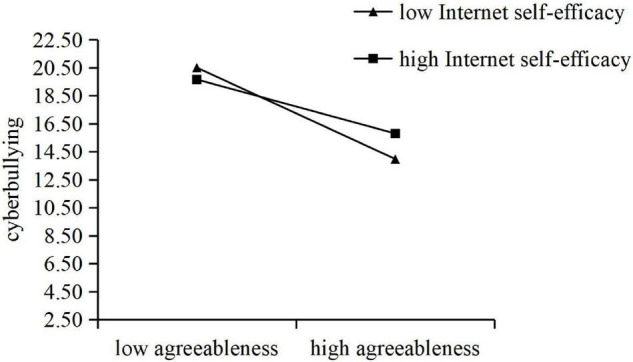
The moderating effect of Internet self-efficacy (ISE) on agreeableness and cyberbullying.

A simple slope test was analyzed. In Figure 1, The result indicated that agreeableness had a significant negative predictive effect on cyberbullying in both the high and low ISE groups. However, compared with the low ISE group (*B* = −0.56, *t* = −8.42, *P* < 0.001), as the score increased (*B* = −0.33, *t* = −5.41, *P* < 0.001), the negative predictive effect of agreeableness on cyberbullying decreased.

### The Moderating Effect of Gender

**FIGURE 2:**
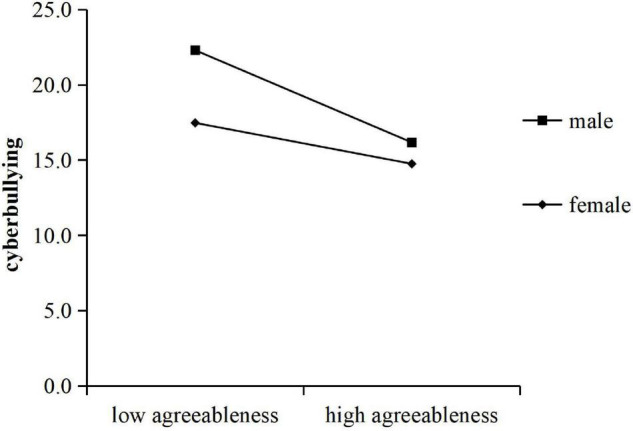
The moderating effect of gender on agreeableness and cyberbullying.

**FIGURE 3:**
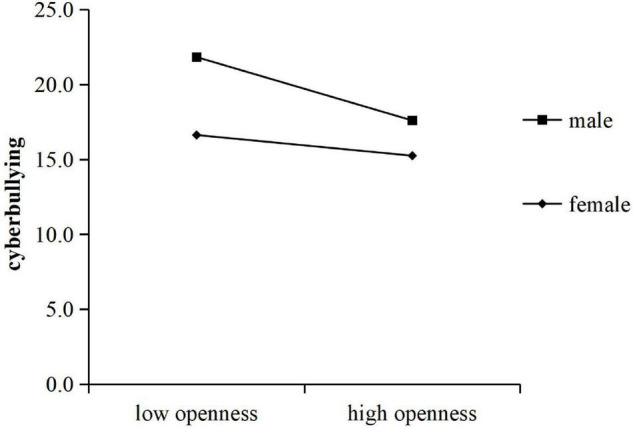
The moderating effect of gender on openness and cyberbullying.

Following the above steps, the moderating effect of gender was tested, and the result showed that there was a significant interaction between agreeableness and gender (*B* = 0.29, *t* = 3.18, *P* < 0.01), with an independent impact of 2% on cyberbullying. The interaction between openness and gender is also significant (*B* = 0.23, *t* = 2.58, *P* = 0.01), with an independent impact of 1% on cyberbullying (Table 3). A simple slope test indicated that, shown in Figure 2, compared with girls (*B* = −0.23, *t* = −3.48, *P* < 0.01), agreeableness of boys had a greater negative effect on cyberbullying (*B* = −0.53, *t* = −8.36, *P* < 0.001). A simple slope test showed that openness had a greater negative effect on cyberbullying in the male group (*B* = −0.35, *t* = −5.63, *P* < 0.001) and a smaller power in the female group (*B* = −0.11, *t* = −1.70, *P* > 0.05). As shown in Figure 3.

**TABLE 3:** The moderating effect of gender.

	Cyberbullying					
	Step 1			Step 2		
	*B*	*t*	*P*	*B*	*t*	*P*
Agreeableness	−0.39	−8.40	<0.001	−0.53	−8.36	<0.001
Gender	−3.13	−5.79	<0.001	−3.12	−5.83	<0.001
Agreeableness × Gender				0.29	3.18	0.002
Δ*R*^2^	0.18		0.02			
Δ*F*	59.13	<0.001	10.11	0.002		
Openness	−0.24	−5.26	<0.001	−0.35	−5.63	<0.001
Gender	−3.77	−6.79	<0.001	−3.77	−6.82	<0.001
Openness × Gender				0.23	2.58	0.01
Δ*R*^2^	0.12			0.01		
Δ*F*	35.96		<0.001	6.63		0.01
						

## Discussion

### The Relationship Between Personality Traits and Cyberbullying Perpetration

There was no significant relation between neuroticism and cyberbullying, unlike what we expected (H1a). The result is consistent with those observed in earlier studies (Semerci, 2017; van Geel et al., 2017; Zhou et al., 2019), but is different from the study of Çelik et al. (2012). Some researchers believed that neurotic individuals are sensitive, vulnerable, anxious, depressed, and impulsive, which makes them more likely to be victims of online bullying (Alonso and Romero, 2017), not perpetrators. As we hypothesized (H1b), agreeableness was negatively associated with cyberbullying, the effect size is medium, according to Cohen (1988). Higher agreeable individuals are altruistic, benignant, and compassionate, which prevents them from bullying others. There was also a significant negative correlation between conscientiousness and cyberbullying, as well as openness and cyberbullying, but the effect size was small. Individuals with high conscientiousness and openness tend to be responsible, restrained, fair, intelligent, and imaginative. Thus, they are less likely to engage in harmful behaviors such as cyberbullying. In addition, the correlation between extraversion and cyberbullying was not statistically significant, which also accords with those earlier observations (van Geel et al., 2017; Zhou et al., 2019). Extroversion, it seems, is not a major predictor of cyberbullying.

### The Moderating Effect of Internet Self-Efficacy and Gender

Internet self-efficacy has played a significantly moderated role in the relationship between agreeableness and cyberbullying, which supported one of our hypotheses (H2b). With the increase of ISE scores, shown in the simple slope analysis, the negative effect of agreeableness on cyberbullying tended to slow down.

High agreeableness is a protective factor for cyberbullying (van Geel et al., 2017; Kowalski et al., 2019), but this protection will be affected by ISE. Rothbart and Ahadi (1994) and Rothbart and Bates (1998) suggested that agreeableness may be a personality trait developed from the temperamental self-regulative system, especially the effortful control, partly serving anger regulation. The increase in agreeableness reflects their increasing ability and willingness to inhibit negative influences and impulses (Wang et al., 2017), However, low-agreeable individuals show impulsivity, reactivity, and negative emotionality (Laursen and Richmond, 2014). They are not thoughtful and do not consider their ability to use the Internet when carrying out cyber attacks. Therefore, individuals with low agreeableness will not be affected by ISE when they engage in cyberbullying. With the increase of agreeableness, their ability and willingness to inhibit impulsivity become stronger, and their cyberbullying behavior decreases. However, if they are confident in their Internet use that engaging in cyberbullying would not put them at risk, their cyberbullying behavior might actually increase compared to low ISE.

This finding confirms that gender moderates the relationship between agreeableness and cyberbullying, which showed that the negative prediction of agreeableness on cyberbullying was more significant in boys. This is consistent with our hypothesis (H3). That is, compared with boys with low agreeableness, boys with high agreeableness were less likely to cyberbully. For the girls, although the overall trend was negative, the degree was weaker than that of the boys. The same moderating effect was found between openness and cyberbullying. Compared to girls, boys have higher levels of cyberbullying. As the openness scores increased, boys’ cyberbullying declined significantly. Therefore, to explore the influence of personality traits on cyberbullying, the moderating effect of gender should be considered. For boys, improving their agreeableness, and openness is beneficial to reducing cyberbullying perpetration.

### Limitations and Practical Implications

There are several limitations in the present study. First, data collection relies on self-report measures, which may result in social desirability bias. Although self-report measurement of cyberbullying has demonstrated validity, future studies could attempt to collect additional information from teachers or peers. Second, counterbalancing technique wasn’t used in this study with respect to the order of administration of the instruments and measures, which may lead to sequence effect. Third, the cross-sectional design is adopted in this study, which only points out the relationship among variables and cannot make causal inferences. Fourth, the participants are undergraduates from Chinese universities and cannot be generalized to other samples.

Despite these limitations, the findings of our study have important implications for intervening in cyberbullying among university students. Firstly, high agreeableness can reduce cyberbullying, therefore, cultivating people’s high agreeableness is important to reduce and intervene in cyberbullying. Secondly, the possible harm of high ISE should not be ignored. Sometimes, over-confidence in net use may make university students unscrupulous in the network environment. Thirdly, More attention should be paid to boys in cyberbullying interventions, not only because they are more likely to participate in bullying and be bullied, but also the protective effect of positive personality traits (such as agreeableness and openness) on cyberbullying is more obvious in this group.

## Data Availability Statement

The raw data supporting the conclusions of this article will be made available by the authors, without undue reservation.

## Ethics Statement

The studies involving human participants were reviewed and approved by the Ethics Committee of Binzhou Medical University. The patients/participants provided their written informed consent to participate in this study.

## Author Contributions

WX designed the work, analyzed the data, and drafted the manuscript. SZ conceived and revised the manuscript. Both authors contributed to the article and approved the submitted version.

## Conflict of Interest

The authors declare that the research was conducted in the absence of any commercial or financial relationships that could be construed as a potential conflict of interest.

## Publisher’s Note

All claims expressed in this article are solely those of the authors and do not necessarily represent those of their affiliated organizations, or those of the publisher, the editors and the reviewers. Any product that may be evaluated in this article, or claim that may be made by its manufacturer, is not guaranteed or endorsed by the publisher.
